# Predictors of Admission in Adult Unscheduled Return Visits to the Emergency Department

**DOI:** 10.5811/westjem.2018.8.38225

**Published:** 2018-09-10

**Authors:** Jake Hayward, Reidar Hagtvedt, Warren Ma, Aliyah Gauri, Michael Vester, Brian R. Holroyd

**Affiliations:** * University of Alberta, Department of Emergency Medicine, Edmonton, Alberta, Canada; † University of Alberta, Alberta School of Business, Edmonton, Alberta, Canada; ‡ Alberta Health Services, Emergency Strategic Clinical Network, Edmonton, Alberta, Canada

## Abstract

**Introduction:**

The 72-hour unscheduled return visit (URV) of an emergency department (ED) patient is often used as a key performance indicator in emergency medicine. We sought to determine if URVs with admission to hospital (URVA) represent a distinct subgroup compared to unscheduled return visits with no admission (URVNA).

**Methods:**

We performed a retrospective cohort study of all 72-hour URVs in adults across 10 EDs in the Edmonton Zone (EZ) over a one-year period (January 1, 2015 – December 31, 2015) using ED information-system data. URVA and URVNA populations were compared, and a multivariable analysis identified predictors of URVA.

**Results:**

Analysis of 40,870 total URV records, including 3,363 URVAs, revealed predictors of URVA on the index visit including older age (>65 yrs, odds ratio [OR] 3.6), higher disease acuity (Canadian Emergency Department Triage and Acuity Scale [CTAS] 2, OR 2.6), gastrointestinal presenting complaint (OR 2.2), presenting to a referral hospital (OR 1.4), fewer annual ED visits (<4 visits, OR 2.0), and more hours spent in the ED (>12 hours, OR 2.0). A decrease in CTAS score (increase in disease acuity) upon return visit also increased the risk of admission (−1 CTAS level, OR 2.6). ED crowding at the index visit, as indicated by occupancy level, was not a predictor.

**Conclusion:**

We demonstrate that URVA patients comprise a distinct subgroup of 72-hour URV patients. Risk factors for URVA are present at the index visit suggesting that patients at high risk for URVA may be identifiable prior to admission.

## INTRODUCTION

In strained healthcare systems globally, there is growing pressure to ensure efficient and high-quality care delivery. Therefore, it is important to develop performance metrics that can be used to monitor care quality and reflect important attributes of patient care. Several quality measures have been proposed and employed in emergency medicine including the number of patients who leave without being seen, ambulance diversion times, total length of stay, and the time delay from a patient’s arrival until being seen by a provider.^1^ This paper explores another performance metric – the unscheduled return visit (URV).

The URV refers to patients who are discharged from the emergency department (ED) and return unexpectedly within a specified time frame. Large, multi-hospital, quality improvement programs have used 72-hour URVs to monitor for adverse events and medical error.^2^ Similarly, in the inpatient setting reimbursement and accreditation programs may penalize hospitals for high rates of readmission for certain medical conditions.^3^ The assumption underlying such surveillance is that the URV represents a potentially avoidable event and may be associated with unsafe or ineffective care. Chart reviews lend support to this idea, revealing links between URVs and missed diagnoses, premature discharge, and inadequate discharge instructions in the ED.^4^–^7^

Existing literature exploring the URV as an ED performance indicator is inconsistent. Published time frames for the URV range from 24 hours to 30 days, and the proportion of URVs that are considered avoidable may be as low as 3% to as high as 32%.^8^,^9^ It is not surprising, then, that the degree of validity and utility of the metric remains unclear. For example, Pham et al. (2011) studied a large national database to find that 72-hour URV patients have similar disease severity, resource utilization, and rates of admission compared to other ED patients.^10^

The distinction between unscheduled return visits with admission (URVA) and unscheduled return visits with no admission (URVNA) may underpin some of the confusion surrounding the URV, as most investigations do not examine URV subgroups. In some guidelines, however, expert consensus recommends using the URVA over the URVNA to monitor ED performance.^1^ This opinion is supported by Hu et al. (2012) who report a stronger link to medical error in URVAs than URVNAs.^11^ In contrast, Sabbatini et al. (2016) found that URVAs were associated with lower mortality in the hospitalized population bringing the metric’s validity into question.^12^ Consequently, the utility of distinguishing between URVAs and URVNAs remains uncertain and many EDs continue to use the URV overall for performance measurement.

Here, we set out to compare URVA and URVNA populations in a large Canadian cohort. We hypothesized that these were distinct groups with different patient and disease factors at their initial, or index, ED visit. We described and compared each population and then evaluated for predictors of URVA.

## METHODS

### Data Source

The Edmonton Zone (EZ) of the Alberta Health Services (AHS) provincial healthcare delivery system contains 10 EDs that capture patient information using standardized data entry (Emergency Department Information System or “EDIS”). Clinical data is entered first by a triage nurse and then by the bedside nurse and attending emergency physician. Our source population was comprised of adult patients (greater than or equal to 17 years) who had a return visit within 72 hours of an index ED visit in the EZ between January 1, 2015, and December 31, 2015. The 72-hour threshold used by AHS for quality assurance is an accepted national standard.^2^ This study was reviewed and approved by the University of Alberta Health Research Ethics Board.

**Population Health Research Capsule**

What do we already know about this issue?

Unscheduled return visits (URVs) are often used as a quality metric in emergency medicine. Some URVs result in admission to hospital (URVAs) whereas others do not (unscheduled return visits with no admission [URVNAs]).

What was the research question?

Are URVAs a distinct high-risk subgroup of URVs compared to URVNAs?

What was the major finding of the study?

URVA patients tend to be older, sicker, and have unique presenting symptoms.

How does this improve population health?

Identifying high-risk patients at emergency department (ED) discharge may help to prevent future hospital admissions. Healthcare administrators can better understand, measure, and improve ED quality of care.

### Patient Selection

We excluded patients from the initial cohort who did not represent a URV. Firstly, patients whose return visit was scheduled or planned were excluded. These patients are flagged as ‘Expected’ in the EDIS system and, for example, might represent a patient who is asked to return to the ED for cast removal or a corneal abrasion recheck. Additionally, we excluded patients whose final disposition was not “Discharged With Approval.” Examples of alternative dispositions include “Left Without Being Seen,” “Left Against Medical Advice” and “Transferred With Approval.” Lastly, frequent ED users were excluded. These patients represent a distinct group with frequent ED use who have an increased risk of URV often attributable to patient-related factors.^13^ We defined frequent users as those patients whose number of ED visits during the study period was in the top 5% of the sample (95^th^ percentile). While there is no universally accepted definition of frequent users, our definition is consistent with that used by other investigators.^14^

### Variable Selection

Study variables were divided into two broad categories: 1) patient related and 2) system related. Patient-related variables included age (17–29, 30–49, 50–64, 65+ years), triage score (1 to 5), change in triage score (Visit 2 – Visit 1), and presenting complaint (according to the Canadian Institute of Health Information Presenting Complaint List).^15^ System-related variables included mode of transport (personal vehicle, air, ambulance, police), hospital type (academic teaching, referral community, and non-referral community), triage time (0700–1459, 1500–2259, 2300–0659), occupancy level (see description below), and total hours spent in the ED (0–4, 4–8, 8–12, >12 hrs).

Triage scoring used the Canadian Triage and Acuity Score (CTAS) measure.^16^ The score is graded from 1 (most acute) to 5 (least acute). Standardized presenting complaints were recorded according to pre-defined CTAS categories, which are comprised of two elements: a broad, system-based descriptor (e.g., “gastrointestinal”) and a more specific symptom (e.g., “abdominal pain”).^17^ We used the symptom for our descriptive analysis and the system-based descriptor for the multivariate analysis. Change in triage score was the only variable that used data from the return visit and was computed as the difference in score between the return and index visits. For example, a score of 4 at the index visit and 2 upon return would result in a change in triage score of −2. Thus, a negative value suggested a deterioration of health status.

Occupancy level was used as a measure of ED crowding. Occupancy level represents the number of patients registered at the time of triage divided by the number of care spaces in that ED; it is expressed as a proportion and was coded as a continuous variable. No single best metric for ED crowding exists; however, occupancy level has been previously used and validated.^18^–^20^

### Statistical Analysis

We performed statistical analysis using statistical software (SAS v9.4, SAS Institute Inc., Cary, NC, USA). For categorical variables, URVA and URVNA populations were compared for similarity using the chi-squared test. For continuous variables, we performed a comparison of means using the t-test. Presenting complaints were ranked and the relative frequencies of the top 10 most frequent in the study cohort are reported; direct pairwise comparisons were not performed. To identify predictors of URVA, a logistic regression was carried out.

## RESULTS

### Population of Study

A total of 470,902 adult ED visits occurred during the study year with an overall admission rate of 12.4%. Of these visits, 40,870 were URVs (return rate of 8.7%). Excluded patients included 3,354 who were “Expected,” 9,263 who were not “Discharged with Approval,”, and 3,171 who were frequent ED users. Of the URVs there were 3,363 URVAs, giving a URV admission rate of 8.2%.

### Descriptive Analysis

Comparison of group means are shown in Table 1. On average, URVA patients were older than URVNA patients (54.5 vs. 44.8 years, p <.0001) with lower CTAS scores (3.0 vs. 3.4, p <.0001). CTAS scores decreased between the index and return visit in the URVA group but not the URVNA group (−0.04 vs. +0.41, p <.0001). URVA patients had fewer ED visits during the study year (5.2 vs. 6.1, p <.0001) and presented slightly earlier in the day (13:54 vs. 14:18, p <.0001). Occupancy level at triage and total hours spent in the ED were higher in the URVA group (158% vs. 144%, p <.0001; 7.0 vs. 4.5 hrs, p <.0001).

Chi-squared tests revealed significant differences between URVA and URVNA patients for the ED type and mode of transport variables (Table 2). Trends suggest that URVA patients are more likely to arrive by ground emergency medical services rather than in private vehicle or ambulatory. Additionally, they are more likely to be seen initially at an academic teaching hospital or referral community center. The most frequent presenting complaints at the index visit are reported in Table 3. The most frequent complaint overall was “abdominal pain,” which occupied a greater proportion in the URVA group. Notable trends included more instances of “shortness of breath” in the URVA group and a higher proportion of “wound checks” and “prescription requests” in the URVNA group.

### Logistic Regression

Predictors of URVA are shown in Table 4. Older age was associated with URVA for all age strata with those over 65 years at particularly high risk of admission (odds ratio [OR] 3.6 [3.2 – 4.0]). Fewer ED annual visits also increased the risk of URVA (0–4 visits, OR 2.0 [1.7 – 2.4]). Patients spending more total hours in the ED were more likely to be admitted when they returned (OR 2.0 [1.7 – 2.4] for >12 hrs). Gastrointestinal symptoms at the index visit conferred 2.7 times the odds of admission, and URVA patients were more likely to initially present at an academic teaching hospital (OR 1.4 [1.2 – 1.5]) or a referral community center (OR 1.4 [1.3 – 1.6]). Higher index-visit triage scores predicted URVA. Compared to the most common CTAS score of 3, a score of 2 was associated with 2.6 times the risk of admission. Furthermore, an increase in disease acuity upon return visit, indicated by a more acute triage score by one level, increased risk of admission by 2.6 times (95% confidence interval [CI] [2.4 – 2.7]). Mode of arrival, time of triage, and occupancy level at the index visit did not emerge as predictors of URVA. Surprisingly, higher occupancy level reduced the odds of admission, albeit to a seemingly negligible degree.

A sensitivity analysis including frequent users of the ED did not significantly alter the results. Predictors of admission remained constant apart from the presenting complaint category – only abdominal pain and general/minor complaints remained predictors. No new risk factors emerged.

## DISCUSSION

The overall URV rate in our study (8.7%) is consistent with estimates from multi-hospital, statewide U.S. data (7.5%).^21^ Hospital-specific data captures only a subset of URVs and therefore often yields lower estimates (e.g. 1.3% – 5.5%).^4^,^22^,^23^ Thus, it is important to use aggregate data to calculate the URV metric. We observed important differences between URVA and URVNA patients. In particular, advanced age was a strong predictor of admission on the repeat ED visit. This finding aligns with previously reported associations between older age and ED boarding time, resource utilization, and mortality.^24^–^26^ Elderly patients have also demonstrated higher rates of 72-hour URV to the ED.^27^–^29^ Our data confirm that older age remains a high-risk feature within the URV population.

We found a robust link between index triage scores and URVA. Existing evidence relating disease severity measures and URVs is conflicting. One study found similar disease acuity in patients admitted to hospital independent of prior ED visit.^12^ Another large retrospective analysis showed that URV patients did not have a higher incidence of vital sign abnormalities compared to the average ED patient.^10^ These studies analyzed disease severity at the return visit, whereas we analyzed the index visit and the change in health status upon return. Therefore, we cannot confirm or refute these findings; however, our results suggest that index triage scores may be an important consideration in risk stratification. Future studies should seek to further evaluate the utility of triage scores, both at index and return visits, in predicting adverse outcomes in URV patients.

The existing literature reporting typical symptom constellations associated with URVs is heterogeneous and inconsistent, varying with study population (e.g., URV vs. URVA) and diagnostic coding systems. Nevertheless, there are a few consistent effects. Gastrointestinal symptoms, and abdominal pain in particular, have been repeatedly linked to URVs.^30^–^36^ Our findings confirm the importance of this presentation, demonstrating a nearly three-fold increase in odds of admission on the repeat visit in those with gastrointestinal symptoms. We also show that patients with respiratory and obstetric/gynecologic complaints are high risk; future subgroup analyses of these complaint categories could reveal specific high-risk disease processes. By focusing on URVA patients, it appears that the incidence of typically low-risk presentations such as wound check, localized swelling, and prescription request are minimized. In turn, the URVA may more accurately reflect a high-risk set of diseases that are clinically challenging on presentation to the ED.

After the exclusion of frequent ED users, fewer annual ED visits predicted URVA in our study. In turn, patients who visit the ED frequently have, on average, a lower risk for admission, perhaps because their presentations reflect patient-related factors such as social instability, or lack of primary care access. In contrast, those who present infrequently may be more likely to be experiencing an acute, rapidly progressive, or severe illness. Consistent with this interpretation is the observation that when URVA patients returned to the ED they demonstrated an average decrease in CTAS score (increased disease acuity) relative to their index visit. URVA patients also spent a longer time in the ED at their initial visit, perhaps indicating more extensive investigations or more complex presentations.

A longer ED length of stay, alternatively, might suggest a more crowded ED. Surprisingly, however, our proxy for ED crowding – occupancy level – was negatively correlated with URVA when other variables were controlled. The explanation for this result is unclear. One possibility is that reduced crowding is associated with high-risk features that were not measured in this study. For example, there is typically less crowding on overnight shifts but also less staffing coverage, increased fatigue, and decreased consulting service and radiology support. Alternatively, the occupancy level metric may not accurately capture ED crowding. For example, “unofficial care spaces” such as hallway stretchers are typically not reported to governing bodies but would alter an ED’s true capacity. Over 70 crowding indicators have been used in the existing literature, none of which are extensively validated.^37^ Despite this limitation, our findings agree with previous authors who have found no association between ED crowding and URVs.^38^–^40^ Future studies should attempt to further delineate the relationship between URVAs and ED crowding.

Future studies should also seek to establish links between URVs (URVAs in particular) and clinically important outcomes. Excess resource utilization associated with the URV should be quantified, including investigations, consultations, and therapies. To clarify the link between URVs and care quality, the relative associations between URVNAs, URVAs, and medical error is important. Ultimately, delineating the risk factors for URVA will drive predictive modelling and clinical decision support systems, which may reduce their occurrence. These findings may also serve to promote awareness of URVA risk factors, allowing clinicians to identify high-risk scenarios at an index visit and alter the chosen disposition.

## LIMITATIONS

Our study’s findings are bolstered by a large sample size taken from all EDs within a large, well-defined geographic region. Thus, we overcame the limitations of publications using hospital-specific data, which may be insensitive to patients who present initially to one ED and return to another. We do recognize, however, that a small proportion of patients may have sought care outside of the ED when they returned to hospital. Our choice of variables was limited by logistic, practical, and technologic constraints, leaving the possibility that confounding effects were unobserved. For example, we were unable to include medical comorbidities or vital signs, which are important patient-related variables. In addition, we have little information about the events that occurred during the ED visits themselves, such as consultations, investigations, and therapies. To effectively assess validity URVs should be linked to mortality and/or morbidity; we were not able to obtain this data using the available database.

Notably, using a 95^th^ percentile cut-off to define frequent users implies a dichotomy where there is likely a continuum. A proportion of patients in the upper range of annual ED visits are likely similar to frequent users. Our sensitivity analysis including “frequent fliers” did not change our results, suggesting that the distinction itself may be artificial or not clinically important. Further studies might better define frequent ED users as a distinct subgroup.

## CONCLUSION

Our work contributes a more detailed understanding of the 72-hour URV ED patient population of an entire health region. We show that measurable variables related to the patient, their disease, and the healthcare delivery apparatus are linked to the risk of admission when a patient returns to the ED. Overall, patients who are admitted upon return are older with fewer annual ED visits. At the index visit, they more often present to large referral hospitals with higher disease acuity, high-risk symptom profiles, and they spend a longer time in the ED. In turn, URVAs represent a high-risk group that is identifiable at initial presentation, and compared to URVNAs or URVs at large, they may be the superior quality metric in emergency medicine.

## Figures and Tables

**Table 1:** Mean comparisons in URVNA and URVA populations.

	URVNA (n = 37,507)	URVA (n = 3,363)	p-value
	
	Mean	Mean	
Age	44.8	54.5	<.0001
ED visits in year	6.1	5.2	<.0001
Hours in ED (hours)	4.5	7.0	<.0001
Time of triage (24-hr clock)	14:18	13:54	<.0001
Occupancy level (%)	144	158	<.0001
Change in triage score	+0.41	−0.04	<.0001
Initial triage score	3.4	3.0	<.0001

*URVNA,* unscheduled return visits with no admission; *URVA*, unscheduled return visits with admission; *ED*, emergency department.

**Table 2:** Frequency distributions for URVNA and URVA populations.

	URVNA (n = 37,507)	URVA (n = 3,363)	p-value
	
	Proportion (%)	Proportion (%)	
ED Type			<.0001
Academic tertiary	19.9	31.0	
Referral community	34.9	41.2	
Non-referral community	45.2	27.8	
Mode of arrival			<.0001
Private vehicle/ambulatory	89.8	72.3	
Ground ambulance	9.3	26.5	
Police	0.41	0.65	
Other	0.05	0.13	

*URVNA,* unscheduled return visits with no admission; *URVA*, unscheduled return visits with admission; *ED*, emergency department.

**Table 3:** Frequent presenting complaints in URVA and URVNA populations.

URVNA (n = 37,507)		URVA (n = 3,363)	
	
Rank	%	Rank	%
1. Abdominal pain	14.8	1. Abdominal pain	21.2
2. Localized swelling	7.7	2. Shortness of breath	5.4
3. Wound check	6.7	3. Pregnancy issues <20 weeks	3.3
4. Pregnancy issues < 20 weeks	4.6	4. Flank pain	2.8
5. Prescription request	3.7	5. Lower extremity pain	2.5
6. Flank pain	3.4	6. Chest pain	2.5
7. Lower extremity pain	3.2	7. Headache	2.0
8. Chest pain	2.5	8. Local swelling	2.0
9. Headache	2.4	9. Wound check	1.0
10. Shortness of breath	2.1	10. Prescription request	0.5
11. Other	49.0	11. Other	57.0

*URVNA,* unscheduled return visits with no admission; *URVA*, unscheduled return visits with admission.

**Table 4:** Factors associated with URVA in logistic regression analysis.

	OR (95% CI)	p-value
Age		
18–30	reference	
30–50	1.3 (1.1 – 1.4)	<0.0001
50–65	1.8 (1.6 – 2.0)	<0.0001
>65	3.6 (3.2 – 4.0)	<0.0001
Triage score		
1	6.6 (3.2 – 13.6)	<0.0001
2	2.6 (2.3 – 2.9)	<0.0001
3	reference	
4	0.3 (0.25 – 0.33)	<0.0001
5	0.1 (0.08 – 0.13)	<0.0001
Change in triage score (−1 point)	2.6 (2.4 – 2.7)	<0.0001
Presenting complaint		
Gastrointestinal	2.2 (1.4 – 3.5)	0.001
Respiratory	1.7 (1.1 – 2.8)	0.03
General and minor	1.7 (1.0 – 2.8)	0.04
Obstetric/gynecologic	1.6 (1.0 – 2.7)	0.05
Minor trauma	reference	
Hours in ED		
0–4	reference	
4–8	1.3 (1.2 – 1.4)	<0.0001
8–12	1.4 (1.3 – 1.7)	<0.0001
>12	2.0 (1.7 – 2.3)	<0.0001
Number of visits in year		
0–4	2.0 (1.7 – 2.4)	<0.0001
4–8	1.9 (1.6 – 2.2)	<0.0001
8–12	1.4 (1.2 – 1.7)	0.003
>12	reference	
Type of hospital		
Academic teaching	1.4 (1.2 – 1.5)	<0.0001
Referral community	1.4 (1.3 – 1.6)	<0.0001
Non-referral community	reference	
Occupancy level (+1%)	0.99 (0.99 – 1.00)	0.02

*OR*, odds ratio; *URVA*, unscheduled return visit with admission; *ED*, emergency department; *CI*, confidence interval.
